# Role of Gene Mutations in Acute Myeloid Leukemia: A Review Article

**DOI:** 10.1055/s-0043-1770768

**Published:** 2023-06-23

**Authors:** Himanshu Singh, Magesh Kumar, Himanshu Kanungo

**Affiliations:** 1 Department of Oral and Maxillofacial Pathology and Oral Microbiology, Index Institute of Dental Sciences, Indore, Madhya Pradesh, India; 2 Department of Periodontics, Index Institute of Dental Sciences, Indore, Madhya Pradesh, India; 3 Department of Orthodontics and Dentofacial Orthopaedics, Index Institute of Dental Sciences, Indore, Madhya Pradesh, India

**Keywords:** Acute myeloid leukemia, gene mutation, genetic alteration

## Abstract

Acute myeloid leukemia (AML) is an immensely heterogeneous disease characterized by the clonal growth of promyelocytes or myeloblasts in bone marrow as well as in peripheral blood or tissue.

Enhancement in the knowledge of the molecular biology of cancer and recognition of intermittent mutations in AML contribute to favorable circumstances to establish targeted therapies and enhance the clinical outcome. There is high interest in the development of therapies that target definitive abnormalities in AML while eradicating leukemia-initiating cells. In recent years, there has been a better knowledge of the molecular abnormalities that lead to the progression of AML, and the application of new methods in molecular biology techniques has increased that facilitating the advancement of investigational drugs.

In this review, literature or information on various gene mutations for AML is discussed. English language articles were scrutinized in plentiful directories or databases like PubMed, Science Direct, Web of Sciences, Google Scholar, and Scopus. The important keywords used for searching databases is “Acute myeloid leukemia”, “Gene mutation in Acute myeloid leukemia”, “Genetic alteration in Acute myeloid leukemia,” and “Genetic abnormalities in Acute myeloid leukemia.”

## Introduction

Acute myeloid leukemia (AML) is a clonal malignancy arising from hematopoietic stem cells. AML is represented by recurrent gene mutations, microRNA deregulations, heterogeneous chromosomal abnormalities, and epigenetic modifications affecting the structure of the chromatin. 1 2 AML advances expeditiously and can be lethal or destructive if not treated. Patients with AML present fatigue, easy bruising, breath shortness, and bleeding. Also, patients show a high possibility of infection, and myeloblasts may be scattered into the skin, gums, and brain. 3 4 5 6

In this review, literature or information on various gene mutations for AML is discussed. English language articles were explored across numerous directories or databases like PubMed, Web of Sciences, Google Scholar, Science Direct, and Scopus. The important keywords used for searching databases were “Acute myeloid leukemia”, “Gene mutation in Acute myeloid leukemia”, “Genetic alteration in Acute myeloid leukemia,” and “Genetic abnormalities in Acute myeloid leukemia.”

### WT1 Mutations

Mutations in the *WT1* gene often result in Wilms tumors because of the lessened DNA binding potential of WT1 protein resulting in the rampant growth and division of cells. *WT1* gene exhibits tumor suppressor function. Various types of research stated that WT1 mutations occur in relatively 9% of cases of AML. Patients of AML with FLT3-ITD mutations, biallelic CEBPα mutations, and PMLRARA fusion show a greater frequency of WT1 mutations. 7 8 9 10

### TP53 Mutations

Mutations in the TP53 gene results in the occurrence of various category of tumors. In 8 to 14% of patients with AML, TP53 mutations have been recognized. 11 12 Mutations and/or deletions of TP53 have been intently linked with complex karyotype AML, resistance to chemotherapy, older age, and overall survival. 2 13

### TET2 Mutations

In 8 to 27% of cases of de novo AML, TET2 mutations have been recognized. 14 15 TET2 catalyzes the modification of 5-methylcytosine to 5-hydroxymethylcytosine in DNA which results in DNA methylation. 16 17 Patients with TET2 mutations show worse prognosis in individuals having NPM1 and FLT3. TET2 is considered to be a tumor suppressor gene in AML. 18 19

### MLL Mutations

The *MLL* gene consists of histone methyltransferase activity (H3K4), and it is positioned on chromosome 11q23. Ten percent cases of adult AML patients show *MLL* gene mutations. 20 21 22 23

### ASXL1/2 Mutations

It is observed that during usual hematopoiesis, ASXL1 behaves as a tumor suppressor gene. Mutations in ASXL1 were recognized in 5 to 11% of patients having AML. In many patients having AML, ASXL1 mutations are either nonsense or frameshift mutations. 24 25 26 27 Mutations in ASXL1 are jointly privileged to FLT3-ITD and NPM1 and participate with IDH1/2, TET2, RUNX1, and EZH2. 28 Currently, ASXL1 mutant protein has been recognized to perform a necessary performance in leukemogenesis and myeloid differentiation in virtue of BAP1. Hence, aiming the BAP1 catalytic activity may be considered a promising therapeutic approach for myeloid malignancies with mutations in ASXL1. 29 30

### DNMT3A Mutations

DNA methyltransferase is encoded by the *DNMT3A* gene. Mutations of DNMT3A were noticed in 15 to 22% of patients having AML. 31 32 But, a large frequency of mutations in DNMT3A has been recognized in CN-AML. It is observed that mutations in DNMT3A are found to be relevant with antagonistic effects in patients that are associated with either FLT3 mutations or the intermediate risk group. 33 34

Mutations in DNMT3A comply with mutations in IDH1/2, FLT3, and NPM1 in AML. 35 When the patient is given extreme doses of daunorubicin, those patients having AML with mutations in DNMT3A or NPM1 demonstrate better overall and relapse-free survival. 36 The treatment of patients having AML with DNMT3A mutation, hypomethylating agents (like decitabine and azacytidine) demonstrates valuable effects. 37 Decitabine accompanying valproic acid demonstrates an advantage in elder patients of an AML patient who were unsuitable for chemotherapy with the least possible toxicity and improved efficacy in the phase-II study. 38 Guadecitabine (SGI-110), an advanced DNMT inhibitor, improved the activeness of decitabine in cancer models of murine. Guadecitabine usage demonstrates the definite clinical response in refractory or relapsed AML cases with acceptable toxicity. 39

### IDH1/2 Mutations

IDH1/2 mutations are considered oncogenic in nature. IDH1/2 mutations demonstrate comprehensive hypermethylation in AML cases. 18 40 41

### EZH2 Mutations

EZH2 is appropriate for the differentiation and maintenance of stem cells. Homozygous mutations of EZH2 were recognized in myeloid malignancies. 42 EZH2 mutations result in worse prognosis and lower relapse-free survival in AML patients. 43 44 OR-S1 and UNC1999 demonstrate a meaningful decrease in clonogenic potentiality and increase in differentiation of MLL-AF9 as well as MLL-AF10 leukemic cells. 45 46

### NPM1 Mutations

NPM1 is a nuclear phosphoprotein that protects the usual cellular function. NPM1 mutations are considered as most persistent mutations that take place in 25 to 35% of AML patients. 47 48 These mutations are answerable for confined NPM1 protein in the cytoplasmic section of the cell. NPM1 mutations are seen high in CN-AML patients. 49 CN-AML patients having wild-type FLT3 and NPM1 mutation show advantageous prognosis and better survival. Likewise, patients having AML with NPM1 mutation in addition to wild-type FLT3 and IDH1/2 mutations show favorable prognosis. 50 51

### RUNX1 Mutations

RUNX1 is a transcription factor that regulates the differentiation and growth of hematopoietic stem and progenitor cells. 52 53 It is intermittently translocated to RUNX1T1 and shows an advantageous prognosis. 54 55 RUNX1 point mutation has been observed in 5 to 13% of cases of AML. RUNX1 mutations can compare with drug resistance and lower long-term survival. 56 57 58 59 According to Laura and their colleagues, they observed that a deficit of wild-type RUNX1 allele has a considerable consequence on the arrangement of gene expression in AML. This research demonstrates that glucocorticoids restrain the OCI-AML3 cell's growth by means of communication with the glucocorticoid receptor. 60

### CEBPα Mutations

CEBPα is a fundamental lineage-specific transcription factor that encourages the gene expression necessary for the differentiation and growth of myeloid progenitors. 61 62 CEBPα mutations are recognized in about 10 to 15% of all cases of AML including CN-AML. CEBPα biallelic mutations are primarily linked with advantageous prognosis. 63 64 65

### FLT3 Mutations

FLT3 is eminently expressed in hematopoietic stem cells and is important for the growth of cells and hematopoietic stem cell differentiation. 66 FLT3 is a transmembrane ligand-activated RTK (receptor tyrosine kinase) that performs a valuable role in the early stages of lymphoid lineage and myeloid growth. FLT3 mutations are observed in nearly 30 to 35% of cases of recently diagnosed AML. 67 68 69 FLT3-TKD-type mutations develop in approximately 7 to 10% of cases of AML, whereas FLT3-ITD-type mutations develop in around 25% of patients of AML. 70 71 Numerous FLT3 inhibitors consist of gilteritinib, crenolanib, sorafenib, sunitinib, quizartinib, and ponatinib.

Gilteritinib is an orally convenient small molecular receptor TKI used in the treatment of AML suppressing FLT3 mutations. Gilteritinib prohibits FLT3 signaling in cells compelling TKD mutation FLT3-D835Y, FLT3-ITD, and double mutant FLT3-ITD-D835Y, by that activating apoptosis. 72

Sunitinib is a small molecule FLT3 inhibitor. Sunitinib has antiangiogenic and antitumor properties. 73 74 75 Sunitinib encourages G1 phase arrest, decreases antiapoptotic, and increases proapoptotic molecule expression in cells of AML. Sunitinib demonstrates synergistic effects with daunorubicin and cytarabine in preventing proliferation and durability of primary AML myeloblasts expressing mutant FLT3-D835V, FLT3-ITD, or FLT3-WT. 76 77

Crenolanib is an influential type I pan-FLT3 inhibitor. Crenolanib is useful in contrary to resistance-conferring TKD and ITD mutations. Crenolanib monotherapy response is temporary and relapse ultimately occurs. Crenolanib does not activate FLT3 secondary mutations. 78

Quizartinib is an influential and selective type 2 FLT3 inhibitor. patients with FLT3-ITD AML are successfully treated with quizartinib. They prohibit FLT3 so that they depress oncogenic drive, which results in tumor cell apoptosis. 79 80

Sorafenib is a multikinase inhibitor. Sorafenib illustrates potential in FLT3+ AML monotherapy. In association with definitive chemotherapy, sorafenib lengthens the survival of a patient with increased toxicity under 60 years of age. 74 81 In phase 2 clinical trial, sorafenib and omacetaxine mepesuccinate proved as a successful therapy for AML patients with a mutation in FLT3-ITD. In another research, sorafenib with exhaustive chemotherapy enhances survival in patients with recently confirmed cases of FLT3-ITD mutated AML. 82 83

### c-KIT Mutations

c-KIT is a receptor tyrosine kinase transmembrane protein. Mutations in c-KIT have been observed in approximately 12 to 46% of cases of AML. 84 85 86 87

### Mutations in Cohesin Complex Members

Cohesin is a protein aggregate that can manage chromosomal segregation. Cohesin complex mutations (RAD21, STAG1, STAG2, SMC1A, and SMC3) have been observed in approximately 13% of patients with AML. 88 Cohesin complex mutations demonstrate the arrangement of collective exclusivity but are accompanied by NPM1, TET2, DNMT3A, and RUNX1 mutations. As SMC1A and STAG2 mutations are found in male patients because these genes are X-linked. Mutations in cohesion complex are vigorously demonstrates unsatisfactory clinical outcome. 89

## Conclusion

The application of oral small-molecule and targeted therapies for AML has rapidly increased in recent years. Regardless of modern clinical progress, AML remains a highly heterogeneous disease with a poor prognosis. Endeavor has aimed on recognizing important metabolic, signaling, and homeostatic pathway that demonstrates potentiality for the advancement of antileukemic drugs. Progress in the development of molecular characterization of AML has furnished noteworthy knowledge for disease observation, diagnosis, and development of therapeutic strategies. Unique therapies for AML like immunotherapies, chemotherapies, and epigenetic and genetic targeted drugs have essentially enhanced patient outcomes. Molecularly targeted therapies have transformed the prospects of treatment of AML and contributed to patients by enhancing survival and condition of life.
